# Non-high density lipoprotein cholesterol/high density lipoprotein cholesterol is L-shaped associated with all-cause mortality and U-shaped with cardiovascular mortality in hypertensive patients

**DOI:** 10.3389/fendo.2025.1490229

**Published:** 2025-03-13

**Authors:** Meiqian Chen, Li Zhang, Qian Liu, Qingxin Gu, Shuhui Yu, Guanzhen Lu

**Affiliations:** ^1^ Department of Internal Medicine-Cardiovascular, Huzhou Central Hospital, Affiliated Central Hospital of Huzhou University, Fifth School of Clinical Medicine of Zhejiang Chinese Medical University, Zhejiang, China; ^2^ School of Medicine and Nursing, Huzhou University, Zhejiang, China

**Keywords:** non-high-density lipoprotein cholesterol to high-density lipoprotein cholesterol ratio, cardiovascular mortality, all-cause mortality, hypertension, lipid

## Abstract

**Background:**

Patients with hypertension may have better survival rates when their lipid metabolism is in balance. The relationship between the novel composite lipid metric, NHHR, and all-cause and cardiovascular mortality in hypertensive patients remains unknown and warrants further investigation.

**Methods:**

We analyzed data from 5,561 hypertensive participants in the National Health and Nutrition Examination Survey (NHANES) spanning from 1999 to 2018. To determine the optimal NHHR cutoff point, we employed a maximum selection statistics approach. Participants were subsequently divided into groups for multivariate weighted Cox regression analysis. The association between NHHR and mortality risk was examined using restricted cubic splines (RCS). To investigate possible variations among different populations, subgroup analysis and interaction tests were carried out. The predictive capability of NHHR for survival outcomes was evaluated using time-dependent receiver operating characteristic (ROC) curves.

**Results:**

Over a median follow-up period of 93 months, patients with hypertension exhibited an all-cause mortality rate of 21.78% and a cardiovascular mortality rate of 7.32%. A markedly elevated risk of cardiovascular death was associated with NHHR levels below 1.66 (HR 1.76; 95% CI 1.31-2.38; P < 0.001), as well as an increased risk of all-cause mortality (HR 1.58; 95% CI 1.31-1.91; P < 0.001). The RCS analysis revealed a U-shaped relationship with cardiovascular mortality (P = 0.0083) and an L-shaped correlation with all-cause mortality (P < 0.0001). Areas under the curve (AUC) for cardiovascular mortality were 0.97, 0.76, and 0.87, and for all-cause mortality were 0.76, 0.84, and 0.80 for the 3-year, 5-year, and 10-year survival periods.

**Conclusions:**

The findings indicate that lower NHHR is associated with an increased risk of both cardiovascular and all-cause mortality, highlighting the critical need for comprehensive lipid management in the clinical management of hypertension. These results suggest that NHHR could serve as a valuable tool for identifying high-risk individuals for mortality, and should be incorporated into routine risk stratification assessments for hypertensive patients. This could more effectively improve patient prognosis and guide personalized treatment strategies.

## Introduction

Adults are diagnosed with hypertension if their systolic blood pressure is at least 130 mmHg and/or their diastolic blood pressure is at least 80 mmHg, as defined by the National Heart, Lung, and Blood Institute (NHLBI) and the Centers for Disease Control and Prevention (CDC) (1). Under this updated criterion, the prevalence of hypertension in the general U.S. population increased from 32.0% (using traditional thresholds) to 46.0% (2). Despite this adjustment, hypertension remains a major contributor to all-cause mortality and is a leading cause of cardiovascular disease and premature death, posing a significant public health challenge (3).

In individuals with hypertension, an inability to adequately regulate blood pressure and cholesterol levels significantly increases the risk of mortality (4). Extensive research has established a link between lipid profiles and cardiovascular risk, revealing that the coexistence of hypertension and dyslipidemia is associated with a heightened risk of cardiovascular disease (CVD). While high-density lipoprotein cholesterol (HDL-C) is traditionally considered protective against cardiovascular events, recent findings suggest that elevated HDL-C levels may paradoxically increase the likelihood of such events (5). Additionally, persistently high levels of non-high-density lipoprotein cholesterol (non-HDL-C) can contribute to the development of atherosclerosis (6). Therefore, evaluating non-HDL-C or HDL-C in isolation may not adequately reflect their true impact on cardiovascular health.

A novel composite lipid marker for atherosclerosis, known as the ratio of non-HDL-C to HDL-C (NHHR), incorporates both HDL-C and non-HDL-C levels, can more comprehensively reflect abnormalities in lipid metabolism (7). According to recent research, NHHR may be a more accurate indicator of insulin resistance, diabetes, metabolic syndrome, and hyperuricemia than conventional lipid markers (8–10). Abnormalities in lipid metabolism are often associated with a higher risk of all-cause mortality, particularly when NHHR levels are low, which may indicate significant fat accumulation and endothelial dysfunction, correlating with a higher risk of cardiovascular mortality (11–13). However, the precise relationship between NHHR levels and the risk of cardiovascular and all-cause mortality in hypertensive populations remains unclear. We hypothesize that NHHR levels are associated with both cardiovascular and all-cause mortality in patients with hypertension, that effective management of NHHR could potentially mitigate mortality risk in this vulnerable population.

## Method

### Data source

**Figure 1:**
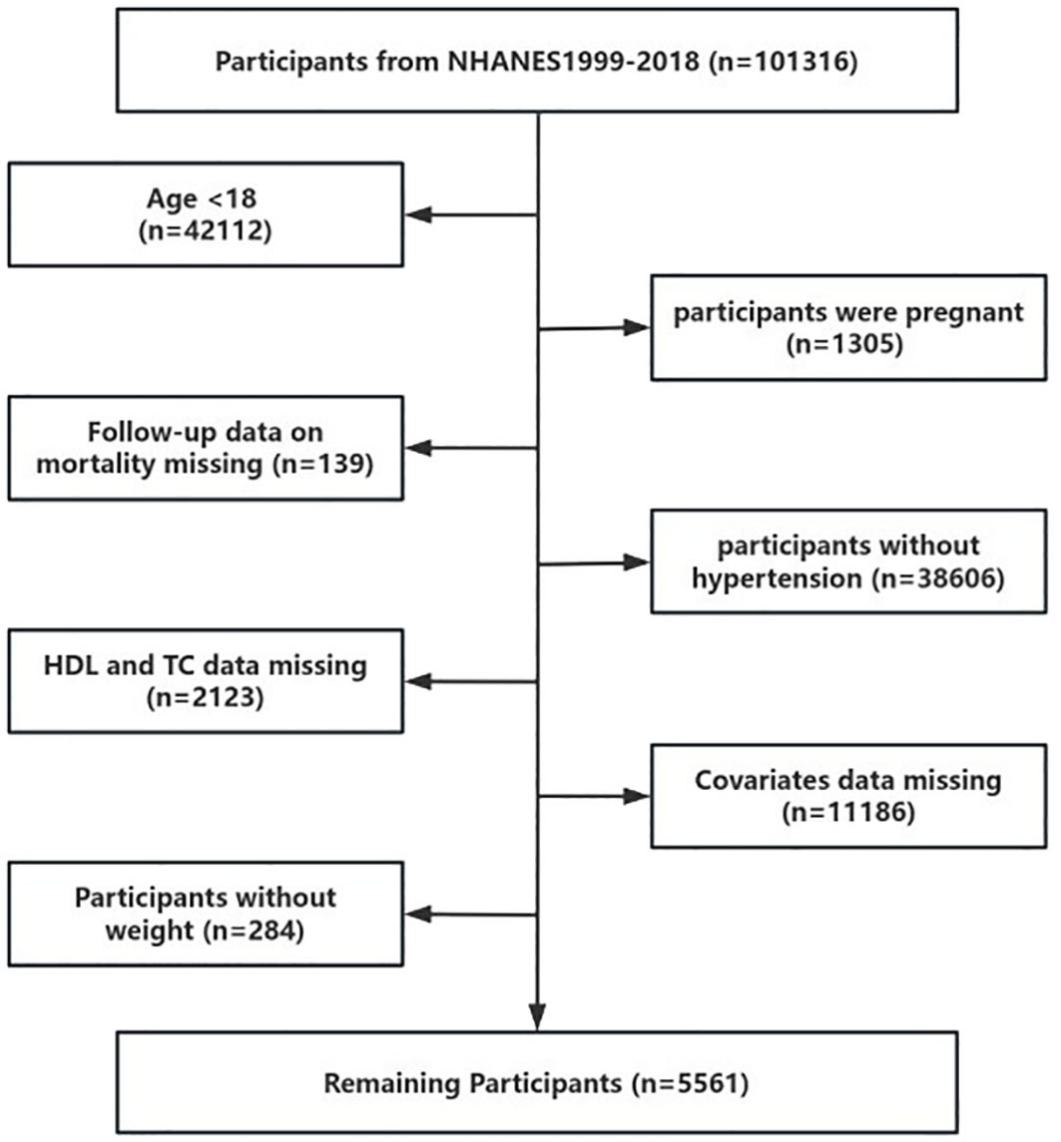
Procedure for enrolling people in research.

Data for this study were sourced from the National Health and Nutrition Examination Survey (NHANES), a comprehensive, nationally representative survey that assesses the nutritional and health status of the U.S. population. Approval for the use of NHANES data was granted by the National Center for Health Statistics Research Ethics Review Board. This investigation utilized data from ten NHANES cycles. Our final analysis included 5,561 participants from 1999 to 2018 (**Figure 1**).

### Exposure variables

In this investigation, the exposure variable was the non-HDL-C/HDL-C ratio (NHHR) (10). Non-HDL-C was subtracted from total cholesterol (TC) to determine HDL-C.

### Covariates

The study incorporated various covariates, including age, sex (male and female), body mass index (BMI) categorized as normal, overweight, or obese, waist circumference (WC), and race (Mexican American, other Hispanic, non-Hispanic White, non-Hispanic Black, and other). Additional covariates included education level (Below high school, High school, Above high school), smoking status, alcohol consumption, history of diabetes, the ratio of family income to poverty (PIR: <1, 1–3, >3), physical activity (0–600, ≥600 Metabolic Equivalent of Energy (MET)-minutes/week), history of cardiovascular disease, low-density lipoprotein cholesterol (LDL-C), triglycerides (TG), blood urea nitrogen (BUN), serum creatinine concentration (Scr), and estimated glomerular filtration rate (eGFR).

Participants were classified into three categories of smoking status: never smokers, former smokers, and current smokers. Alcohol consumption was categorized into four groups: never (<12 drinks in a lifetime), former (≥12 drinks in the previous year but no alcohol in the previous year, or those who exceeded 12 drinks over their lifetime but abstained in the prior year), light to moderate drinkers (less than 1 drinks per day for women and less than 2 for men), and heavy drinkers (≥1 drinks per day for women and ≥2 for men).

Cardiovascular disease (CVD) histories included physician-reported diagnoses of congestive heart failure, angina, stroke, myocardial infarction, and coronary heart disease. eGFR was calculated using the Chronic Kidney Disease Epidemiology Collaboration equation (eGFR_CKD-EPI_) (14).

### Clinical outcomes

All-cause mortality is the study’s primary endpoint, while cardiovascular mortality is its secondary goal. The follow-up period terminated on the death date or December 31, 2019, whichever came first in the study.

### Statistical analysis

Given the complexity of the NHANES design, sample weights provided by the National Center for Health Statistics (NCHS) were incorporated into the statistical analysis.

The “maxstat” package is used to perform Maximum Selective Rank Statistic, determining the optimal threshold of NHHR and categorizing individuals into low and high NHHR groups. It is suitable for continuous variables and can provide a critical point with the highest survival probability in survival outcome variables, facilitating clinical interpretation (15).

Baseline characteristics were compared between lower and higher NHHR groups. Continuous variables were assessed using Analysis of Variance (ANOVA) or Kruskal-Wallis tests, while categorical variables were analyzed using chi-square tests.

In hypertensive individuals, a multivariable weighted Cox proportional hazards model was utilized to elucidate the relationships between the NHHR and both cardiovascular and all-cause mortality. Three sets of models were developed: the crude model included only NHHR; Model 1 adjusted for age, gender, race, BMI, WC, education level, PIR, and marital status; while Model 2 further incorporated smoking status, alcohol consumption, physical activity, history of CVD, diabetes, Scr, BUN, and eGFR. Additionally, a four-knot restricted cubic spline (RCS) logistic regression was employed to explore the nonlinear associations between NHHR and mortality outcomes, enabling a more nuanced understanding of the relationship between NHHR levels and risk of death. If the RCS analysis indicated a nonlinear relationship, an assessment of the threshold effect was conducted.

Subgroup analyses, stratified by age, gender, BMI, smoking status, alcohol consumption, physical activity, history of cardiovascular disease, and diabetes, were carried out to examine the relationship between NHHR and all-cause and cardiovascular mortality. Interaction tests were employed to evaluate heterogeneity in these relationships across various subgroups.

The predictive accuracy of NHHR for 3-year, 5-year, and 10-year survival outcomes was assessed using receiver operating characteristic (ROC) curves generated by the “timeROC” software. All analyses were conducted using R version 4.2.3 and EmpowerStats, with a P-value of less than 0.05 considered statistically significant.

## Results

### Participants’ initial characteristics

**Figure 2:**
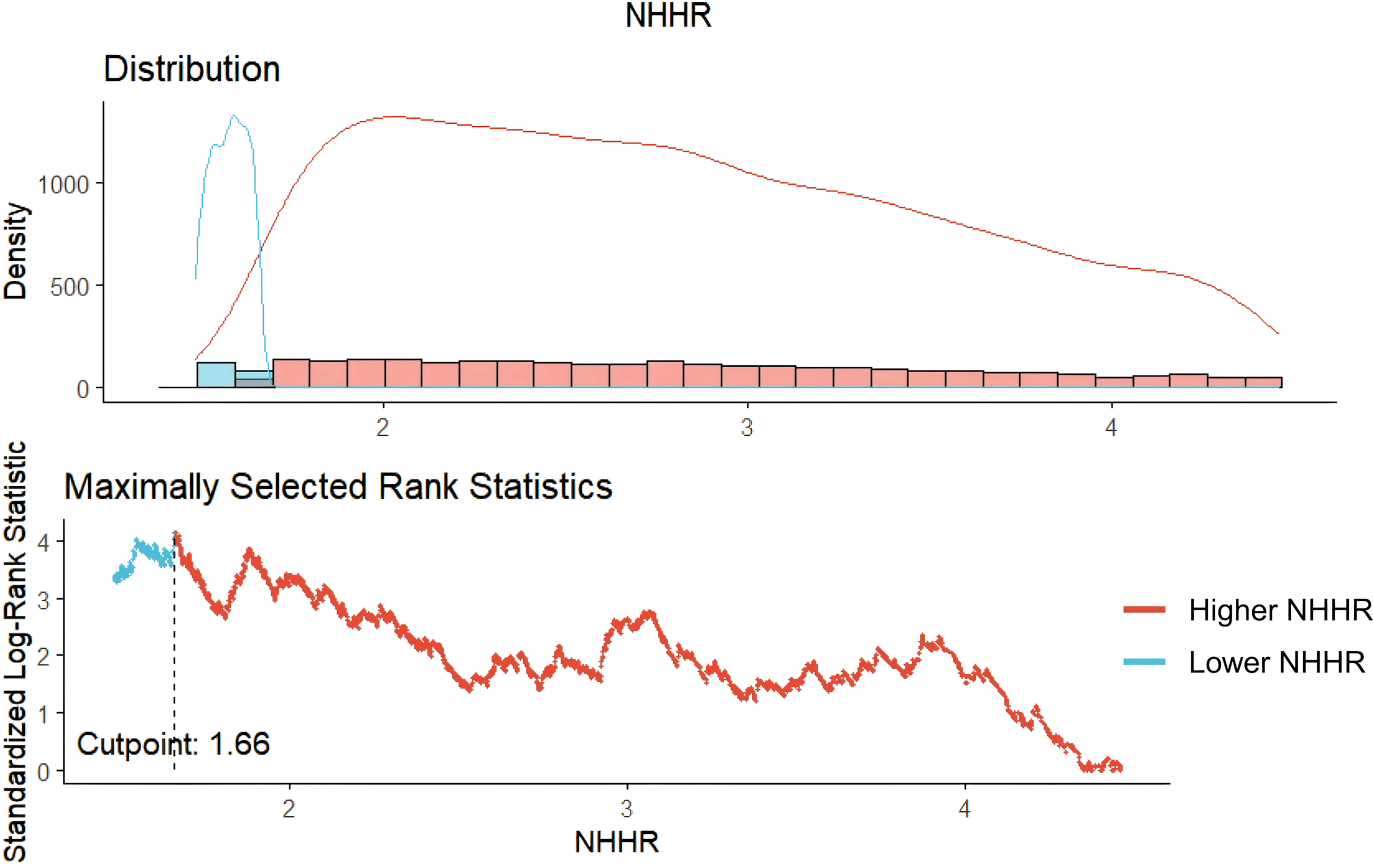
Cut-off value for NHHR.

The optimal cutoff point for NHHR was determined to be 1.66, categorizing participants with NHHR > 1.66 into the Higher NHHR group and those with NHHR ≤ 1.66 into the Lower NHHR group (**Figure 2**). The study comprised 5561 hypertension patients, 4,721 of whom were in the Higher NHHR group and 840 in the Lower NHHR group, as **Table 1** illustrates. The average NHHR level in the Higher NHHR group was 3.17 mmol/L, while the average NHHR level in the Lower NHHR group was 1.32 mmol/L.

**Table 1:** Characteristics of participants.

Characteristic	Overall	Higher	Lower	*p-value*
	N = 5561	N = 4721	N = 840	
NHHR	2.92 (0.02)	3.17 (0.02)	1.32 (0.01)	<0.001
Age (years)	56.73 (0.28)	56.04 (0.32)	61.07 (0.68)	<0.001
Gender, %				<0.001
Male	48.75% (48.43%)	50.56% (50.53%)	38.57% (35.36%)	
Female	51.25% (51.57%)	49.44% (49.47%)	61.43% (64.64%)	
Race, %				<0.001
Mexican American	12.79% (4.88%)	13.54% (5.03%)	8.57% (3.97%)	
Other Hispanic	7.63% (3.82%)	8.05% (4.08%)	5.24% (2.22%)	
Non-Hispanic White	47.96% (72.47%)	48.85% (73.18%)	42.98% (68.07%)	
Non-Hispanic Black	24.26% (12.91%)	22.30% (11.86%)	35.24% (19.50%)	
Other	7.37% (5.92%)	7.27% (5.86%)	7.98% (6.23%)	
Education, %				0.3
Below high school	26.96% (18.16%)	27.43% (18.57%)	24.29% (15.62%)	
High school	25.00% (26.50%)	25.04% (26.35%)	24.76% (27.39%)	
Above high school	48.05% (55.34%)	47.53% (55.08%)	50.95% (56.99%)	
PIR, %				0.9
<1	18.74% (12.29%)	18.96% (12.23%)	17.50% (12.67%)	
1-3	44.22% (38.46%)	44.33% (38.55%)	43.57% (37.89%)	
>3	37.04% (49.25%)	36.71% (49.22%)	38.93% (49.44%)	
Marital, %				0.054
Married	55.49% (60.06%)	56.51% (60.89%)	49.76% (54.85%)	
Single	39.13% (34.06%)	38.06% (33.50%)	45.12% (37.55%)	
With partner	5.38% (5.88%)	5.42% (5.61%)	5.12% (7.59%)	
BMI, %				<0.001
Normal	18.00% (17.64%)	15.46% (14.85%)	32.26% (35.07%)	
Overweight	32.96% (32.06%)	33.28% (32.35%)	31.19% (30.26%)	
Obese	49.04% (50.30%)	51.26% (52.80%)	36.55% (34.67%)	
Physical activity, %				0.5
0 to <600 MET	52.40% (48.42%)	52.49% (48.17%)	51.90% (49.99%)	
≥600 MET	47.60% (51.58%)	47.51% (51.83%)	48.10% (50.01%)	
Drink, %				0.001
Never	29.74% (25.33%)	29.82% (25.60%)	29.29% (23.63%)	
Former	16.29% (13.20%)	16.39% (13.23%)	15.71% (13.01%)	
Mild to Moderate	9.46% (9.71%)	9.87% (10.40%)	7.14% (5.38%)	
Heavy	44.51% (51.77%)	43.91% (50.77%)	47.86% (57.98%)	
Smoking, %				0.8
Never	49.20% (48.45%)	48.78% (48.25%)	51.55% (49.73%)	
Former	32.94% (32.82%)	33.15% (32.93%)	31.79% (32.11%)	
Current	17.86% (18.73%)	18.07% (18.82%)	16.67% (18.16%)	
Diabetes, %	22.15% (17.69%)	21.69% (17.69%)	24.76% (17.70%)	0.9
History of CVD, %	21.30% (19.00%)	20.50% (18.00%)	26.20% (23.00%)	0.01
WC, cm	105.71 (0.28)	106.88 (0.30)	98.35 (0.71)	<0.001
TG,mmol/L	1.52 (0.02)	1.62 (0.02)	0.88 (0.02)	<0.001
LDL-C,mmol/L	2.95 (0.02)	3.09 (0.02)	2.07 (0.03)	<0.001
HDL-C,mmol/L	1.37 (0.01)	1.28 (0.01)	1.93 (0.03)	<0.001
Non-HDL-C,mmol/L	3.65 (0.02)	3.83 (0.02)	2.48 (0.03)	<0.001
Scr,umol/L	83.00 (1.00)	83.00 (1.00)	83.00 (1.00)	0.3
BUN,mmol/L	5.37 (0.04)	5.35 (0.04)	5.48 (0.10)	0.5
eGFR,mL/min/1.73m2	86.00 (0.00)	87.00 (1.00)	83.00 (1.00)	0.002

Continuous variables are reported as means with standard errors (SE), while categorical variables are presented as unweighted percentages and weighted percentages. MET, Metabolic Equivalent of Energy.

Patients in the Lower NHHR group were more likely to be female, older, non-Hispanic Black, and heavy drinkers compared to those in the Higher NHHR group (P<0.05). Additionally, the Lower NHHR group exhibited a slightly higher prevalence of cardiovascular disease, along with lower levels of WC, TG, LDL-C, eGFR, Non-HDL-C, and a slightly higher HDL level (P<0.05).

### Association between all-cause mortality and NHHR in hypertensive patients

**Figure 3:**
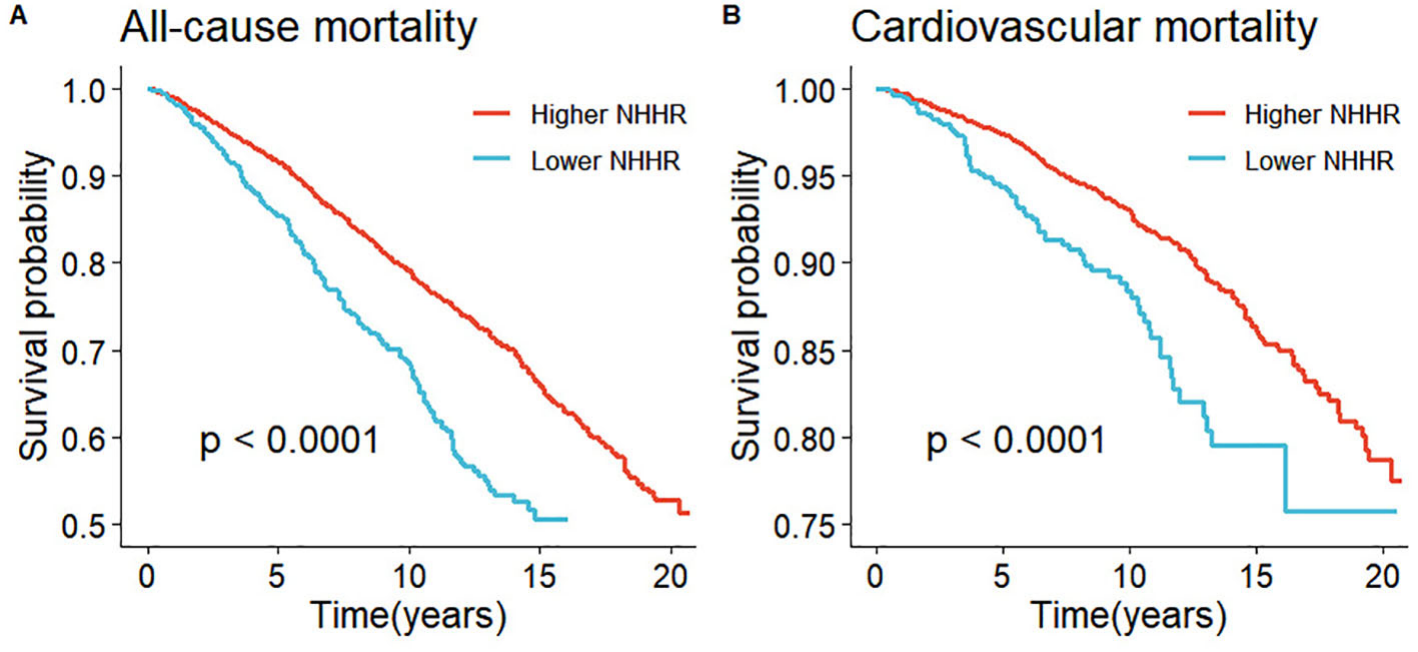
Kaplan-Meier survival analysis results. **(A)** represented all-cause mortality and NHHR and **(B)** represented cardiovascular mortality and NHHR.

1,211 deaths occurred among 5,561 participants over a median follow-up of 93 months. Survival curve analysis revealed that individuals in the Lower NHHR group experienced a significantly lower survival rate compared to those in the Higher NHHR group (P < 0.0001) (**Figure 3A**). In the crude model, all-cause mortality also increased with higher levels of NHHR (HR 1.97, 95% CI: 1.64-2.36, P<0.001) (**Table 2**). After adjusting for confounding variables, Model 1 had an HR of 1.62 (95% CI: 1.35-1.94, P < 0.001), and Model 2 had an HR of 1.58 (95% CI: 1.31-1.91, P < 0.001). This indicates that for every unit increase in NHHR level, the risk of all-cause mortality increased by 62% and 58%, respectively (**Table 2**).

**Table 2:** The relationships between NHHR and mortality in hypertension.

Characteristic	Crude model		Model 1		Model 2	
	HR (95% CI)	*p*	HR (95% CI)	*p*	HR (95% CI)	*p*
*All-cause mortality*						
Higher NHHR	Ref		Ref		Ref	
Lower NHHR	1.97(1.64-2.36)	< 0.001	1.62(1.35-1.94)	< 0.001	1.58(1.31-1.91)	< 0.001
*Cardiovascular mortality*						
Higher NHHR	Ref		Ref		Ref	
Lower NHHR	2.24(1.71-2.93)	< 0.001	1.84(1.36-2.49)	< 0.001	1.76(1.31-2.38)	< 0.001

Crude model: unadjusted model

Model 1: Adjusted for age, gender, race, BMI, WC, education level, PIR, and marital status.

Model 2: Adjusted for age, gender, race, BMI, WC, education level, PIR, marital status, smoking, alcohol consumption, physical activity, history of cardiovascular disease, diabetes, Scr, BUN, and eGFR.

**Figure 4:**
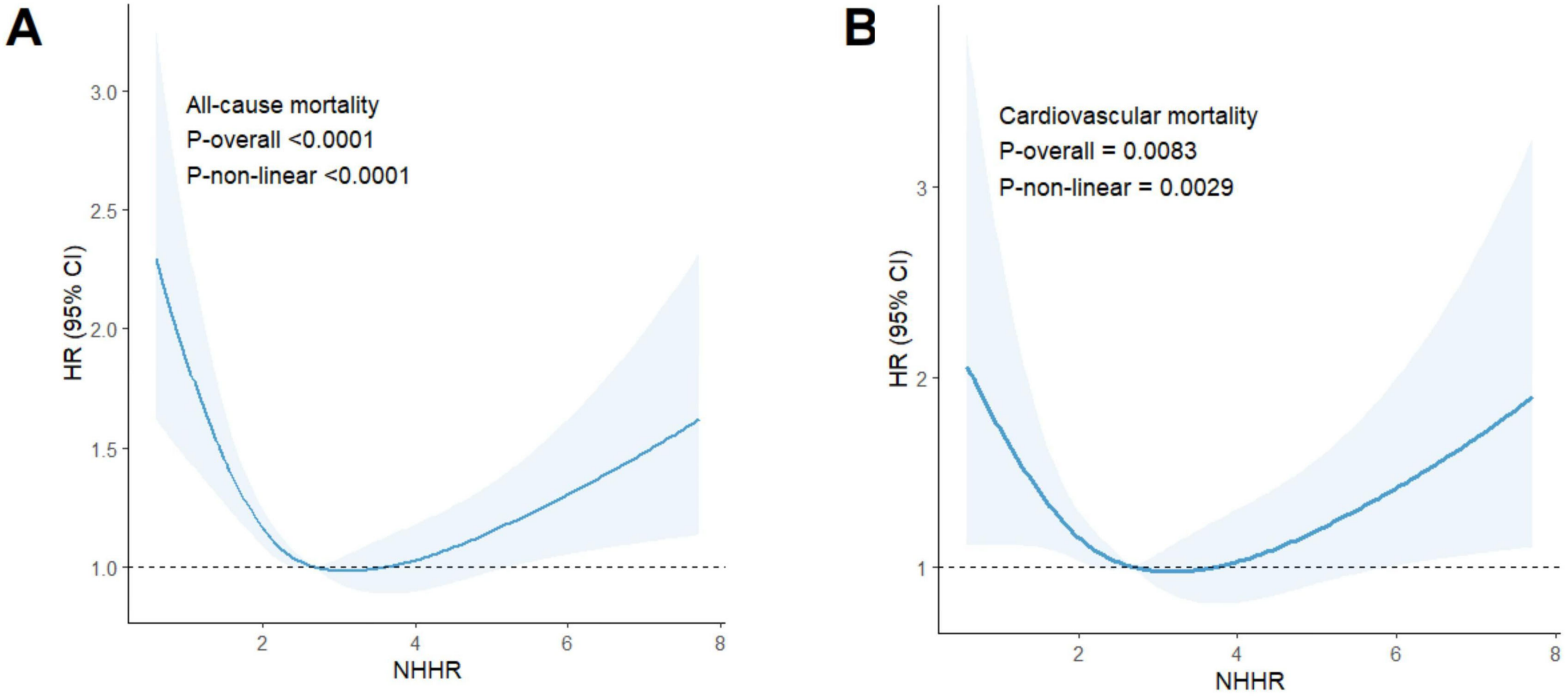
Results of RCS analysis. **(A)** represented all-cause and NHHR mortality, and **(B)** represented cardiovascular mortality and NHHR.

In individuals with hypertension, NHHR levels were strongly associated with all-cause mortality (P-overall < 0.0001), demonstrating an L-shaped relationship (P-non-linear < 0.0001) (**Figure 4A**). The RCS analysis identified a critical point at an NHHR level of 2.99 As detailed in **Table 3**, below this threshold, each unit increase in NHHR was associated with a 21% reduction in the risk of all-cause mortality (HR: 0.79, 95% CI: 0.71-0.88, p < 0.0001). Conversely, above this cutoff, each unit increase in NHHR was linked to a 11% increase in risk (HR: 1.11, 95% CI: 1.03-1.19, P = 0.0045).

**Table 3:** Threshold effect analysis of the NHHR on all-cause and cardiovascular mortality in participants with hypertension.

Characteristic	All-cause mortality		Cardiovascular mortality	
	HR (95% CI)	*P value*	HR (95% CI)	*P value*
Total	0.96(0.92-1.02)	0.1822	1.00(0.91-1.09)	0.9978
Segmented cox proportional hazards model				
Inflection point	2.99		3.17	
NHHR<Inflection point	0.79(0.71-0.88)	<0.0001	0.85(0.72-1.00)	0.0496
NHHR ≥ Inflection point	1.11(1.03-1.19)	0.0045	1.14(1.01-1.30)	0.0340
P for Log-likelihood ratio		<0.001		0.022

No significant interactions were observed between NHHR and all-cause mortality across subgroups stratified by age, gender, smoking status, alcohol consumption, BMI, physical activity, diabetes, or history of cardiovascular disease (interaction p > 0.05) (**Table 4**).

**Table 4:** The correlations between NHHR and death among hypertensive patients were analyzed by subgroup.

Characteristics	All-cause mortality				Cardiovascular mortality			
	Higher NHHR	Lower NHHR			Higher NHHR	Lower NHHR		
		HR (95% CI)	P value	P interaction		HR (95% CI)	P value	P interaction
Age				0.151				0.103
<60 years	Ref	1.03 (0.87-1.22)	0.721		Ref	1.12 (0.92-1.37)	0.256	
60+ years	Ref	0.90 (0.84-0.97)	0.004		Ref	0.91 (0.82-1.02)	0.103	
Gender				0.474				0.167
Male	Ref	0.81 (0.75-0.88)	<0.001		Ref	0.79 (0.66-0.94)	0.007	
Female	Ref	0.85 (0.73-0.99)	0.04		Ref	0.92 (0.79-1.08)	0.304	
BMI				0.875				0.926
Normal	Ref	0.93 (0.80-1.09)	0.388		Ref	0.95 (0.77-1.18)	0.657	
Overweight	Ref	0.81 (0.71-0.92)	0.001		Ref	0.75 (0.60-0.94)	0.013	
Obese	Ref	0.92 (0.81-1.05)	0.215		Ref	0.94 (0.77-1.16)	0.582	
Drink				0.437				0.684
Never	Ref	0.87 (0.76-1.01)	0.062		Ref	0.86 (0.73-1.01)	0.074	
Former	Ref	0.88 (0.78-1.01)	0.063		Ref	1.01 (0.81-1.27)	0.918	
Mild-Moderate	Ref	0.59 (0.45-0.77)	<0.001		Ref	0.50 (0.32-0.80)	0.003	
Heavy	Ref	0.80 (0.68-0.95)	0.009		Ref	0.79 (0.58-1.09)	0.15	
Smoking				0.882				0.387
Never smoker	Ref	0.86 (0.73-1.00)	0.053		Ref	0.82 (0.70-0.96)	0.013	
Former smoker	Ref	0.81 (0.72-0.91)	<0.001		Ref	0.87 (0.69-1.09)	0.222	
Current smoker	Ref	0.88 (0.77-1.00)	0.045		Ref	0.95 (0.74-1.22)	0.675	
Physical activity				0.098				<0.001
0 to <600 MET	Ref	0.82 (0.73-0.92)	0.001		Ref	0.77 (0.67-0.88)	<0.001	
≥600 MET	Ref	0.90 (0.80-1.00)	0.055		Ref	1.06 (0.90-1.25)	0.464	
History of CVD				0.711				0.135
No	Ref	0.85 (0.76-0.96)	0.01		Ref	0.81 (0.69-0.96)	0.014	
Yes	Ref	0.87 (0.80-0.96)	0.004		Ref	0.97 (0.83-1.12)	0.642	
Diabetes				0.14				0.233
No	Ref	0.82 (0.74-0.92)	<0.001		Ref	0.83 (0.73-0.93)	0.002	
Yes	Ref	0.93 (0.82-1.07)	0.317		Ref	1.01 (0.75-1.35)	0.961	

### Relationship between hypertensive patients’ cardiovascular mortality and NHHR

Among the 1211 reported deaths among participants, 407 were attributed to cardiovascular diseases. Survival curve analysis demonstrated that individuals in the Lower NHHR group exhibited a significantly lower survival rate compared to those in the Higher NHHR group (P < 0.0001) (**Figure 3B**). In the crude model, cardiovascular mortality increased with higher levels of NHHR (HR 2.24, 95% CI: 1.71-2.93, P < 0.001) (**Table 2**). After adjusting for confounding variables, Model 1 had an HR of 1.84 (95% CI: 1.36-2.49, P < 0.001), and Model 2 had an HR of 1.76 (95% CI: 1.31-2.38, P < 0.001). This suggests that for every unit increase in NHHR level, the risk of cardiovascular mortality increased by 84% and 76%, respectively (**Table 2**).

The risk of cardiovascular mortality was significantly correlated with NHHR levels (P-overall = 0.0083) and exhibited a U-shaped relationship (P-non-linear = 0.0029) (**Figure 4B**). The RCS analysis identified a critical turning point at an NHHR level of 3.17. Below this threshold, NHHR was inversely associated with the risk of cardiovascular mortality. As detailed in **Table 3**, each unit increase in NHHR below this cutoff was linked to a 15% reduction in the risk of cardiovascular death (HR: 0.85, 95% CI: 0.72-1.00, P = 0.0496). Conversely, above this cutoff, each unit increase in NHHR was linked to a 14% increase in risk (HR: 1.14, 95% CI: 1.01-1.30, P = 0.0340).

Furthermore, no significant interactions were observed between NHHR and cardiovascular mortality across subgroup analyses stratified by age, gender, smoking status, alcohol consumption,BMI, diabetes, or history of cardiovascular disease (interaction p > 0.05) (**Table 4**). Conversely, a significant interaction effect was observed between NHHR and cardiovascular mortality in the subgroup analysis of physical activity (interaction p < 0.001).

### ROC curve analysis of NHHR and mortality with hypertension

**Figure 5:**
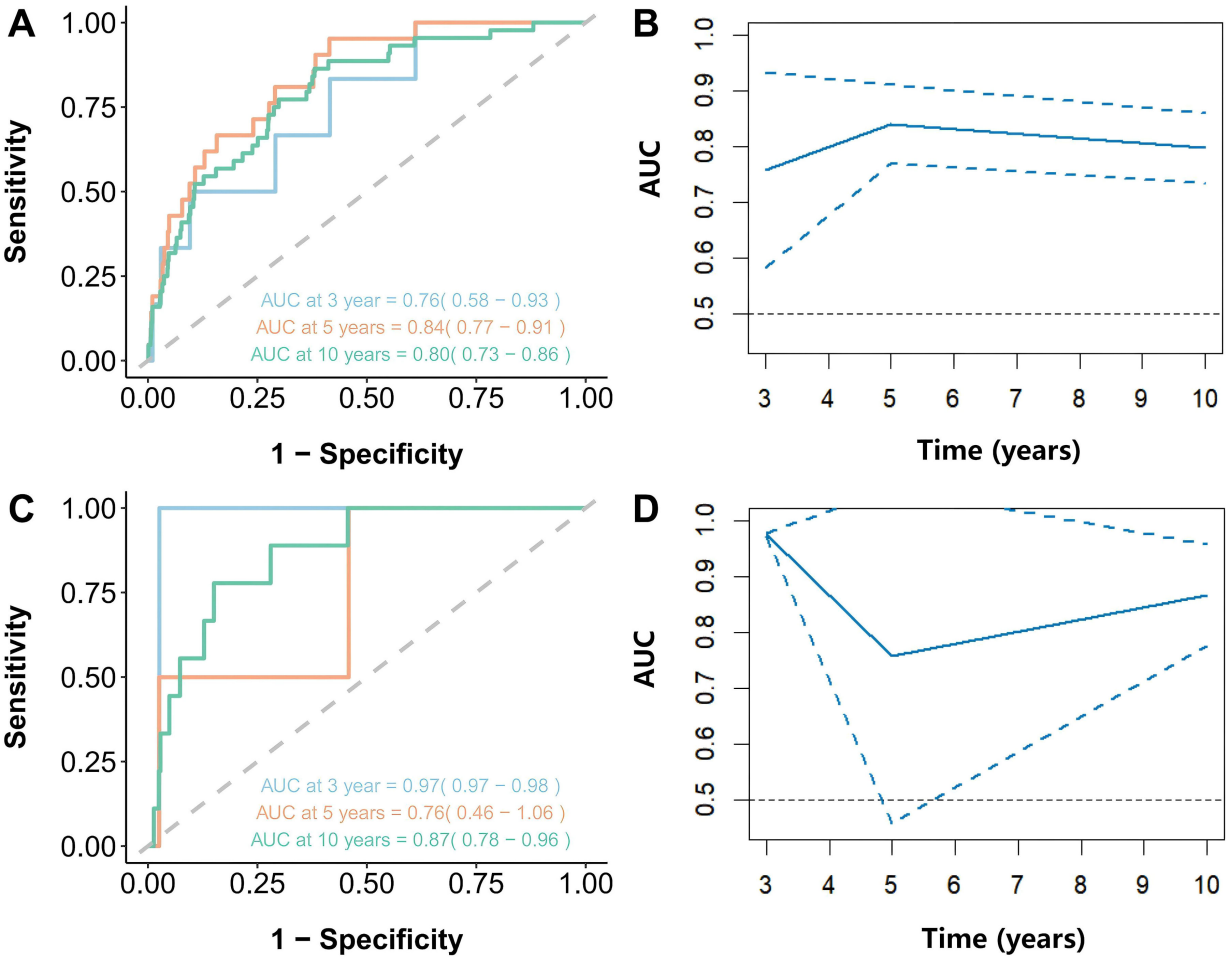
Time-dependent ROC curves and time-dependent AUC values (with 95% confidence band) of the NHHR for predicting mortality. **(A, B)** represented all-cause mortality and NHHR, and **(C, D)** represented cardiovascular mortality and NHHR.

As shown in **Figures 5A, B**, the results showed that the NHHR’s area under the curve (AUC) for predicting all-cause mortality at three, five, and ten years was, respectively, 0.76 (95% CI: 0.58-0.93), 0.84 (95% CI: 0.77-0.91), and 0.80 (95% CI: 0.73-0.86). The AUC values for cardiovascular mortality were 0.97 (95% CI: 0.97-0.98), 0.76 (95% CI: 0.46-1.06), and 0.87 (95% CI: 0.78-0.96) at three, five, and ten years, respectively (**Figures 5C, D**). These findings imply that in individuals with hypertension, NHHR may be a useful predictor of both short- and long-term all-cause mortality as well as cardiovascular death.

## Discussion

This study explored the relationship between NHHR and mortality outcomes in a large, prospective cohort of American patients with hypertension. Among 5,561 hypertensive individuals, NHHR demonstrated a robust association with both cardiovascular and all-cause mortality, underscoring its potential as a prognostic tool for assessing survival outcomes in this population over varying time horizons.

Lipids play a crucial role in cellular stability. Alterations in lipid levels can disrupt cellular integrity and growth, affecting cellular involvement in tissue repair, and thereby increasing the risk of infection, mortality, and disease (16, 17). Studies showed that aberrations in lipid levels are individually and strongly linked to a higher risk of myocardial infarction, heart failure, ischemic stroke, cardiovascular death, and all-cause death (18). Reducing lipid variability during hypertension management may also aid in controlling hypertension and improving cardiovascular mortality and morbidity (19). NHHR may represent a comprehensive measure of lipid changes. Our findings indicated that NHHR may be a useful biomarker for estimating the probability of death in hypertensive individuals. NHHR could serve as an indicator that reflects lipid metabolic abnormalities more effectively than traditional cholesterol levels. The NHHR’s function as an independent predictor of cardiovascular and all-cause death in diabetes individuals provides additional evidence of the study’s indirect support (20).

At baseline, the group with lower NHHR exhibited a larger percentage of older adults, female, and lower levels of TG in the hypertensive population. This evidence is supported by previous research conducted by Cheng et al. (21). Our findings indicate that the relationship between NHHR and all-cause mortality follows an L-shaped trajectory, with intermediate NHHR values associated with the lowest mortality risk. This observation aligns with previous research showing that, within hypertensive populations, intermediate levels of HDL-C correspond to the lowest risk of all-cause mortality (22).Further analysis revealed that excessively low NHHR values significantly elevate the risk of all-cause mortality. Studies have established that low non-HDL cholesterol levels, whether in the general population or among individuals with hypertension, correlate with an increased adjusted risk ratio for all-cause mortality (21, 23). The following mechanisms may help explain this phenomenon: (1) At appropriate HDL-C concentrations, NHHR acts as a protective factor, while very high HDL-C concentrations result in lower NHHR levels. This may be due to genetic variations that exacerbate the harmful effects associated with high risks of disease and mortality, such as mutations in CETP, ABCA1, and LIPC (5, 24). (2) At low NHHR levels, the fraction of HDL particles (HDL-P) rises. Under normal physiological conditions, HDL particles exhibit protective properties, including facilitating reverse cholesterol transport, possessing antioxidant and anti-inflammatory effects, and providing endothelial protection (25). An increased proportion of HDL-P may lead to a loss of these protective functions, rendering them dysfunctional. A prospective cohort study from the UK Biobank supports our findings in this regard (26). The specific mechanisms involved require further investigation. It is crucial to maintain optimal NHHR levels in this population since hypertension individuals who have both high and low NHHR levels are linked to an increased risk of mortality. This suggests that clinicians should pay close attention to changes in NHHR levels when devising treatment plans and follow-up care for hypertensive patients.

In patients with hypertension, NHHR demonstrated a U-shaped relationship with cardiovascular mortality. Previous studies have established that dyslipidemia is one of the primary factors contributing to the increased risk of atherosclerosis in patients with hypertension (27). In patients with single-vessel, double-vessel, or multi-vessel coronary artery disease, NHHR levels are higher compared to the normal group (28). Elevated NHHR levels are significantly associated with an increased incidence of major adverse cardiovascular events in coronary artery disease patients. Cheng et al. reported a U-shaped association between non-HDL cholesterol and mortality in hypertensive individuals, corroborating our findings; they observed that cardiovascular mortality was lower at non-HDL-C levels <130 mg/dL and higher at levels ≥220 mg/dL (21). Similar patterns have been observed in populations with chronic kidney disease (29). From the standpoint of non-HDL-C levels, persistently elevated non-HDL-C can increase NHHR, thereby heightening the risk of CVD in hypertensive patients and further elevating non-HDL-C levels. Our results are consistent with findings from several studies (6, 30, 31), which suggest that abnormal non-HDL-C concentrations, when combined with hypertension, contribute to atherosclerosis through mechanisms such as chronic inflammation, vascular remodelling, endothelial dysfunction, and altered arterial compliance. Conversely, HDL-C levels typically decline as non-HDL-C levels increase, with low HDL-C levels linked to an elevated risk of cardiovascular events (32). Moreover, increased NHHR in hypertensive patients may be associated with prolonged QRS duration, leading to widened QRS complexes, a common characteristic in individuals with potential heart disease (33). Furthermore, extremely high HDL-C levels have been associated with an increased risk of cardiovascular mortality, possibly due to their impact on the pro-inflammatory and anti-inflammatory functions of the immune system (34). Therefore, achieving optimal NHHR and blood pressure values in hypertensive individuals may contribute to mitigating the risk of cardiovascular diseases.

In our study, certain factors such as age, sex, smoking status, alcohol consumption, BMI, diabetes, or history of cardiovascular disease did not exhibit significant interactions with NHHR and the all-cause or cardiovascular mortality in hypertensive patients. This lack of significance may be attributed to the possibility that these factors themselves operate through alternate mechanisms, such as alcohol-induced hepatic steatosis or metabolic disturbances caused by insulin resistance in diabetes, or they may be confounded by various diseases, indirectly impacting overall mortality. These mechanisms and those by which higher or lower NHHR levels affect the body may not entirely overlap (35, 36). However, the significant interaction between physical activity and the cardiovascular mortality of hypertensive patients in relation to NHHR may be due to the close relationship between physical activity and cardiovascular health (37). Physical activity has the potential to enhance cardiovascular function in hypertensive patients, improve endothelial function, reduce inflammation, and enhance cardiac metabolic health (38). For hypertensive patients, low levels of physical activity may significantly increase their risk of cardiovascular mortality, exacerbating the negative impact of NHHR and consequently leading to higher cardiovascular mortality rates (37).

Maintaining the NHHR of hypertensive patients at an ideal level (neither too high nor too low) is an important goal in cardiovascular health management. A suitable NHHR level contributes to balancing cholesterol metabolism, reducing the risk of cardiovascular events, and promoting the overall health of hypertensive patients. A high NHHR may indicate a higher cardiovascular risk for patients, making it crucial to reduce their cardiovascular risk as a primary goal (34). Encouraging patients to adopt healthy lifestyles, such as balanced diets, moderate exercise, smoking cessation, and limited alcohol consumption, can promote cholesterol metabolism balance (38). When necessary, the use of lipid-lowering medications like statins can help control cholesterol levels and decrease cardiovascular risk (39). Conversely, a low NHHR may reflect lower HDL-C levels or higher Non-HDL-C levels, emphasizing the need to adjust lipid metabolism balance and increase HDL levels. It is advisable for patients to further improve their lifestyles by increasing physical activity, selecting healthy fats, increasing dietary fiber, among other strategies, to raise HDL-C levels (40). Healthcare institutions may consider incorporating NHHR into the assessment guidelines and follow-up monitoring of hypertensive patients, integrating it with other clinical indicators such as blood pressure, blood sugar, and lipid levels for a comprehensive evaluation. This approach enables timely adjustments to medication treatment plans, recommends personalized lifestyle interventions, and ultimately reduces cardiovascular risk and improves the overall health status of patients.

## Strengths and limitations

To our knowledge, this study is pioneering in examining the association between NHHR and both cardiovascular and all-cause mortality in individuals with hypertension. Furthermore, our findings were robustly supported by data derived from the large, representative prospective cohort study NHANES (1999-2018). Despite this novel contribution, several limitations must be acknowledged. Firstly, as an observational study, NHANES does not allow for the inference of causality between NHHR and mortality outcomes. Secondly, our investigation was limited to the association between NHHR and cardiovascular as well as all-cause mortality and did not explore the role of NHHR in mortality from other causes, such as cancer. Thirdly, self-reported clinical data were used to make the diagnosis of hypertension, which does not provide clear diagnostic criteria or detailed stratification of hypertension severity; therefore, mortality rates among patients with varying levels of hypertension warrant further investigation. Fourthly, considering the variation in the predictive accuracy of cardiovascular mortality at different time points, future research could focus on optimizing the NHHR prediction model by integrating additional biomarkers or clinical indicators to enhance the accuracy and reliability of long-term predictions. Fifth, NHHR demonstrates varying optimal thresholds depending on the statistical methods employed. Therefore, it is crucial to consider clinical contexts when selecting suitable cutoff points for subsequent research and application.

## Conclusion

The analysis revealed an L-shaped relationship between NHHR and all-cause mortality among hypertensive patients, indicating that lower NHHR levels are associated with increased mortality risk. Conversely, NHHR exhibited a U-shaped correlation with cardiovascular mortality. This suggests that while NHHR may be a robust predictor of both short-term and long-term survival, the nature of its association with mortality risks is complex and warrants further investigation. To clarify the processes by which NHHR affects death rates in hypertensive patients, more prospective validation and investigation is necessary.

## Data Availability

The original contributions presented in the study are included in the article/supplementary material. Further inquiries can be directed to the corresponding authors.
